# Hollow Threats: Transnational Food and Beverage Companies’ Use of International Agreements to Fight Front-of-Pack Nutrition Labeling in Mexico and Beyond

**DOI:** 10.34172/ijhpm.2020.146

**Published:** 2020-08-10

**Authors:** Eric Crosbie, Angela Carriedo, Laura Schmidt

**Affiliations:** ^1^School of Community Health Sciences, University of Nevada Reno, Reno, NV, USA.; ^2^Ozmen Institute for Global Studies, University of Nevada Reno, Reno, NV, USA.; ^3^World Public Health Nutrition Association, London, UK.; ^4^Philip R. Lee Institute for Health Policy Studies, University of California San Francisco, San Francisco, CA, USA.; ^5^Department of Anthropology, History and Social Medicine, University of California San Francisco, San Francisco, CA, USA.

**Keywords:** Food Industry, Nutrition Policy, International Trade, Mexico, Latin Countries

## Abstract

In October 2019, the Mexican government reformed its General Health Law thus establishing the warning approach to front-of-pack nutrition labeling (FOPNL), and in March 2020, modified its national standard, revamping its ineffective FOPNL, one preemptively developed by industry actors. Implementation is scheduled for later in 2020. However, the new regulation faces fierce opposition from transnational food and beverage companies (TFBCs), including Nestlé, Kellogg, Grupo Bimbo, Coca-Cola, PepsiCo through their trade associations, the National Manufacturers, American Bakers Associations, the Confederation of Industrial Chambers of Mexico and ConMéxico. Mexico, as a regional leader, could tip momentum in favor of FOPNL diffusion across Latin America. But the fate of the Mexican FOPNL and the region currently lies in this government’s response to three threats of legal challenges by TFBCs, citing international laws and guidelines including the World Trade Organization (WTO), Codex Alimentarius, and the North American Free Trade Agreement (NAFTA)/US-Mexico-Canada Agreement (USMCA). In this perspective, we argue that these threats should not prevent Mexico or other countries from implementing evidence-informed policies, such as FOPNLs, that pursue legitimate public health objectives.

 In 2016, Chile became the first country in Latin America to adopt a directive interpretive front-of-pack nutrition labeling (FOPNL) with warning labels, which provides simplified nutritional information on packaged foods and beverages.^1^ This warning FOPNL approach adopted improved the use and understanding of nutrition information and the purchasing decisions by consumers, yielding healthier choices.^1,2^ Following the FOPNL adopted in Chile, sugary beverage and cereal purchases decreased by 25% and 9%, respectively.^1,2^ Peru and Uruguay have since adopted a similar approach that a host of other countries in the region of the Americas are currently deliberating (eg, Brazil, Canada).

 In October 2019, the Mexican government reformed its General Health Law thus establishing the warning approach to FOPNL, and in March 2020, modified its national standard, revamping its ineffective FOPNL, one preemptively developed by industry actors.^3^ Implementation is scheduled for later in 2020. However the new regulation faces fierce opposition from transnational food and beverage companies (TFBCs), including Nestlé, Kellogg, Grupo Bimbo, Coca-Cola, and PepsiCo through their trade associations, the National Manufacturers, American Bakers Associations, the Confederation of Industrial Chambers of Mexico and ConMéxico.^4-6^ Mexico, as a regional leader, could tip momentum in favor of FOPNL diffusion across Latin America. But the fate of the Mexican FOPNL and the region currently lies in this government’s response to three threats by TFBCs citing international laws. We argue that these industry threats of legal challenges should not prevent Mexico or other countries from implementing evidence-informed policies that pursue legitimate public health objectives.

## Industry Threats to Sue Over Trade Violations in the World Trade Organization

 The World Trade Organization (WTO) Technical Barriers to Trade (TBT) Agreement aims to prevent regulations, certification procedures, testing and standards (eg, marketing restrictions) from posing unnecessary obstacles to international trade.^7^ TFBCs through their trade associations allege that Mexico’s revamped FOPNL creates unnecessary obstacles to trade by violating Article 2.2 and Article 2.4^4,5^ (Table).

**Table:** TFBCs and Trade Associations’ Threats of Legal Challenges to FOPNL in Mexico: Potential Responses and Supporting Evidence

**International Agreement**	**TFBCs and Trade Associations’ Threats of Legal Challenges**	**Analysis and Potential Response**	**Supporting Evidence**
WTO TBT	FOPNL unnecessarily restricts trade. - Article 2.2 (technical regulations should not create unnecessary obstacles to trade). - Article 2.4 (where relevant international standards exist Members should use them as a basis for their national technical regulations except when such international standards would not effectively fulfill the legitimate objectives pursued).^4,5^	While TBT Agreement cautions against any regulation that unnecessarily restrict trade, it recognizes that each WTO Member has the basic right to implement measures to achieve legitimate policy objectives, such as the protection of human health and safety.^7^	- Similar trade concerns were raised in Chile, Peru and Indonesia in 2013, Ecuador in 2014, and Uruguay in 2019,^8^ but these countries have moved forward with FOPNL. - WTO member states have successfully argued for FOPNL in WTO TBT Committee discussions as ‘providing consumers with sufficient information about the food which they consume and reducing non-communicable diseases;’ ‘provide consumers with information so as to make appropriate dietary choices and reduce the risk of diet-related NCDs;’ and ‘empower consumers to make an informed choice in order to foster effective competition and consumer welfare.’^8^
WTO TRIPS	FOPNL would restrict its trademarks under the WTO TRIPS where the labeling regulation also provides restriction to the use of persuasive elements, such as cartoon characters, on packaged foods required to carry FOPNL warnings.		Similar trade law concerns were raised in Chile, but the country has moved forward with FOPNL.^8^
Codex	FOPNL is inconsistent with international standards of the Codex Alimentarius Commission (Codex Guidelines) and countries should wait until Codex develops FOPNL guidelines.	- There are no existing Codex guidelines specific to FOPNL-as provisions in Codex on supplementary nutrition information is what the FOPNL guideline work has grown out of, thus it is not possible to be inconsistent with these guidelines. - Codex establishes “minimum standards”/a floor and countries may choose to exceed these standards to protect the health of their populations from health risks. - If Codex guidelines are developed in the future, it is not mandatory to comply with them under trade law.	Similar challenges referring to Codex were made in Chile and Uruguay but both countries have moved forward with FOPNL.
NAFTA	Chapter 11 allows foreign investors (eg, corporations) to directly challenge FOPNL under Investor-State Dispute Settlement through the International Centre for Settlement of Investment Disputes.^9^ - FOPNL is an infringement on trademarks due to the removal of graphics or logos on packaging.	Analogous arguments under similar provisions in other investment agreements were made against tobacco packaging and labeling and both domestic and legal courts have ruled against this argument as trademark law protects the owner from infringement (others using their trademark), but does not give them a right to use the trademark in any context. - FOPNL can also be justified on a health basis, which may be appropriate to limit the use of trademarks.	In 2010 and 2011, Philip Morris International sued Uruguay and Australia respectively to challenge tobacco packaging and labeling laws but lost both in domestic and international courts where they had to pay millions of dollars in legal fees.^10,11^
USMCA	- Article 11.4 allows for wider, enforceable language on the recognition of national public or private standardization bodies as relevant international standards. This could extend to accepting voluntary standards (eg, corporate standards that have been developed in the US) as equivalent to Codex standards for the purpose of developing national regulations.^12^	Current Codex Guideline work on FOPNL proposes general guidance, rather than a specific FOPNL to be used. Codex processes are slow and the outcome of this work and its eventual legal status is still unclear. -Article 9.4: Sanitary or phytosanitary measures which conform to relevant international standards, guidelines, and recommendations are deemed to be necessary to protect human, animal, or plant life or health, and presumed to be consistent with the relevant provisions of this Chapter, Chapter 2 (National Treatment and Market Access for Goods), which relate to the use of sanitary or phytosanitary measures, and Article XX(b) of the GATT 1994 as incorporated into Article 32.1 (General Exceptions). -Article 9.6: Each Party has the right to adopt or maintain the sanitary and phytosanitary measures necessary for the protection of human, animal or plant life or health.	The USMCA came into force in July 2020 but Codex Guideline work on FOPNL is still pending.

Abbreviations: NAFTA, North American Free Trade Agreement; TBT, Technical Barriers to Trade; TRIPS, Trade-Related Aspects of Intellectual Property Rights; USMCA, United States-Mexico-Canada Agreement; WTO, World Trade Organization; FOPNL, front-of-pack nutrition labeling; TFBCs, transnational food and beverage companies.

 This represents a simple delay tactic by TFBCs because these concerns have already been raised in the WTO’s TBT Committee concerning FOPNL in Chile, Peru and Indonesia (2013), Ecuador (2014) and Uruguay (2019).^8^ While the TBT Agreement cautions against any regulation that unnecessarily restricts trade, it recognizes that each WTO Member has a basic right to protect human health.^7^ Governments have proceeded with their FOPNL policies having justified them in the TBT Committee on the grounds that they empower consumers, increase knowledge, improve dietary choices and reduce the risk of non-communicable diseases.^8^

 TFBCs have also argued that Mexico’s FOPNL restricts trademarks protected by the WTO Agreement on Trade-Related Aspects of Intellectual Property Rights because of its requirement for labels to remove persuasive elements, such as cartoon characters on the packages of products. Chile has successfully used the above arguments here, allowing it to proceed with implementing such provisions with its FOPNL legislation.^13^ Court precedents exist supporting similar arguments made in relation to tobacco packaging: trademark law protects the owner from infringement (others using their trademark), but does not grant the right to use the trademark in any context.^14,15^

## Industry Efforts to Leverage Codex Alimentarius Standards

 Codex Alimentarius is a Food and Agriculture Organization/World Health Organization (WHO) international food standards program that also provides international guidelines for nutrition labelling and can be referenced in trade forums. Codex standards are widely accepted and often become ‘de facto’ national standards but should not be a barrier for countries proposing to implement stronger FOPNL policies.^16^

 TFBCs argue that Mexico’s FOPNL law is inconsistent with international standards (including Codex),^4,5^ which are recognized in WTO’s TBT Article 2.4 and Article 2.9 (If a measure is not in accordance with international standards, or no relevant standard exists, members shall notify other members, provide information and allow time for comment).^7^ Here, TFBCs are essentially arguing that Codex is the only international standard on the matter and place an internationally binding ceiling on the stringency of FOPNL laws implemented by nation states. Yet Codex does not provide guidance on the details of national policies.^8^ It in fact establishes minimum voluntary standards, or a floor, on national measures that ensure food safety.^17^ Chile, Peru, Ecuador, and Uruguay have all moved forward with their FOPNL laws by making these arguments. Uruguay and Mexico have used the Nutrient Profile Model of the Pan American Health Organization published in 2016, as well as national and international evidence, to craft their FOPNL policies.

## Efforts to Manipulate Regional Trade Agreements

 Chapter 11 of the North American Free Trade Agreement (NAFTA), a regional trade agreement between the United States, Mexico and Canada, allowed foreign investors (eg, TFBCs) to directly challenge national regulations, including public health policies that impact their investments through Investor-State Dispute Settlement in the International Centre for Settlement of Investment Disputes.^9^ Under NAFTA and other foreign investment treaties, TFBCs could try to challenge Mexico’s FOPNL law in international courts, although these challenges are unlikely to succeed given failed attempts in the context of tobacco control. In 2010, Philip Morris International sued Uruguay over its tobacco warning label policy by arguing it defied a bilateral investment treaty. The company lost and wound up paying $8 million for Uruguay’s legal costs.^10^ Philip Morris Asia similarly sued Australia over tobacco packaging under a bilateral investment treaty and also lost, paying millions in costs.^11^

 In November 2018, the three NAFTA parties renegotiated the treaty, creating the United States-Mexico-Canada Agreement (USMCA) which came into force on July 1, 2020, replacing NAFTA. In 2018, leaked drafts of a proposed annex to the USMCA revealed an American-introduced provision that would have prevented any warning symbol, shape or color that ‘inappropriately denotes that a hazard exists from consumption of the food or nonalcoholic beverages.’^18^ This language was dropped following media reports of the leak, although the attempt underscores how aggressively TFBCs seek to chill the spread of FOPNL.^18^ The USMCA’s final language, however, (Article 11.4) allows for wider, enforceable language on the recognition of relevant international standards (ie, Codex guidelines). This could result in the US putting pressure on Mexico to adopt its weak labeling standards, or potentially weak standards established by Codex at some future time.^12^ Potential new Codex work, however, is in the early stages and not all work by Codex necessarily ends with the adoption of a standard or guideline; the work could be discontinued as well.

 The USMCA appears to remove TFBC’s ability to directly challenge national measures, particularly those that protect public health, including nutrition policies in international courts, although corporations in five other economic sectors retain this right (oil and natural gas, power generation, telecommunications, transportation services, and some infrastructure).^12^ This further reduces the possibility of a successful legal challenge to FOPNL in Mexico.

## Conclusion

 TFBCs are attempting to block the WHO’s 2013-2020 Global Action Plan for Prevention and Control of non-communicable disease recommendation of mandatory interpretive FOPNL,^19^ thereby containing diffusion of this public health innovation in Mexico and Latin America. However, international legal challenges are much easier and cheaper to threaten than to carry, much less, to win. TFBCs prefer to avoid any costly legal battles that they are likely to lose. At best, TFBCs will likely delay, not block, implementation of the new Mexican FOPNL. The Mexican executive branch has expedited implementation by setting clear deadlines for the FOPNL regulation guidelines with the support of national and international experts. Mexico should continue this approach despite hollow threats by industry and other countries in the region should do the same.

## Acknowledgement

 We thank Robert Eckford for his legal feedback provided for this perspective.

## Ethical issues

 Not applicable.

## Competing interests

 Authors declare that they have no competing interests.

## Authors’ contributions

 EC conceptualized the study, collected the data and prepared the first and subsequent drafts of the Perspective. EC, AC, and LS contributed to revisions of the article.

## Authors’ affiliations

^1 ^School of Community Health Sciences, University of Nevada Reno, Reno, NV, USA. ^2^Ozmen Institute for Global Studies, University of Nevada Reno, Reno, NV, USA. ^3^World Public Health Nutrition Association, London, UK. ^4^Philip R. Lee Institute for Health Policy Studies, University of California San Francisco, San Francisco, CA, USA. ^5^Department of Anthropology, History and Social Medicine, University of California San Francisco, San Francisco, CA, USA.

## Supplementary files


Supplementary file 1 contains the Spanish translation of the paper.
