# Supplementary material for: Hollow Threats: Transnational Food and Beverage Companies’ Use of International Agreements to Fight Front-of-Pack Nutrition Labeling in Mexico and Beyond
Source: Int J Health Policy Manag. 2020 Aug 10;11(6):722–5. doi: 10.34172/ijhpm.2020.146 (PMC9309911; doi:10.34172/ijhpm.2020.146)
Supplement: Supplementary file 1 — contains the Spanish translation of the paper. [file ijhpm-11-722-s001.pdf]

IJHPM
International Journal of Health Policy and Management

The authors submitted the Spanish translation and the IJHPM Production Manager just reproduced it. This translation was not peer-reviewed. Only the original article, submitted in English, was peer-reviewed. Thus, the English version should serve as reference for this article.

Amenazas vacías: Uso de acuerdos internacionales por las empresas transnacionales de alimentos y bebidas para combatir el etiquetado frontal de los alimentos en México y más allá

Eric Crosbie[1,2], Angela Carriedo[3], Laura Schmidt[4,5]

Resumen

En octubre de 2019 el gobierno mexicano reformó la Ley General de Salud, estableciendo el etiquetado de advertencia (EA) como el nuevo esquema de etiquetado frontal nutrimental de alimentos (EFNA), y en marzo de 2020 la norma oficial mexicana fue modificada con los lineamientos para reformular el etiquetado frontal de alimentos vigente, uno que ha mostrado ser ineficaz, y fue desarrollado en colaboración con actores de la industria. La implementación está programada para finales de 2020. Sin embargo, la nueva regulación enfrenta una oposición feroz de las compañías transnacionales de alimentos y bebidas (CTAB), que incluyen Nestlé, Kellogg, Grupo Bimbo, Coca-Cola y PepsiCo a través de sus asociaciones comerciales, la *National Association of Manufacturers*, la *American Bakers Associations*, la Confederación de Cámaras Industriales de México y el Consejo Mexicano de la Industria de Bienes y Servicios (ConMéxico). México, como líder regional, podría dar un impulso a favor de replicar el EA en América Latina. Pero el destino del etiquetado frontal mexicano y el de la región actualmente radica en la respuesta de este gobierno a tres amenazas de acciones legales por parte de las CTAB, citando leyes y directrices internacionales que incluyen a la Organización Mundial del Comercio (OMC), el Codex Alimentarius y el Tratado de Libre Comercio de América del Norte (TLCAN)/El Tratado entre México, Estados Unidos y Canadá (TMEC). Desde esta perspectiva, sostenemos que estas amenazas no deberían impedir que México u otros países implementen políticas basadas en evidencia, como lo es el etiquetado frontal de advertencia para los alimentos envasados, cuyo objetivo legítimo es la salud pública.

Palabras clave: industria alimentaria, política alimentaria, comercio internacional, México, países latinos

En 2016, Chile se convirtió en el primer país de América Latina en adoptar un etiquetado frontal nutrimental de alimentos (EFNA) directivo e interpretativo, con símbolos de advertencia, que proporciona información nutrimental simplificada de los alimentos y bebidas envasados.1 Este etiquetado frontal de advertencia (EFA) mejoró el uso y la comprensión de la información nutrimental y las decisiones de compra por parte de los consumidores, resultando en elecciones más saludables.1,2 A través de la adopción del EFA en Chile, las compras de bebidas azucaradas y cereales disminuyeron en un 25% y un 9%, respectivamente.1,2 Perú y Uruguay han adoptado un enfoque similar desde entonces, y otros países de la región de las Américas actualmente debaten la adopción de dicho

[1] School of Community Health Sciences, University of Nevada Reno, Reno, NV, USA.
[2] Ozmen Institute for Global Studies, University of Nevada Reno, Reno, NV, USA.
[3] World Public Health Nutrition Association, London, UK.
[4] Philip R. Lee Institute for Health Policy Studies, University of California San Francisco, San Francisco, CA, USA.
[5] Department of Anthropology, History and Social Medicine, University of California San Francisco, CA, USA.

sistema de etiquetado (por ejemplo, Brasil y Canadá).

En octubre de 2019, el gobierno mexicano reformó la Ley General de Salud, estableciendo como obligatorio el EFA para los alimentos empacados, y en marzo de 2020, modificó la norma oficial mexicana con los lineamientos, para reformular el EFNA vigente, uno que ha mostrado ser ineficaz, y fue desarrollado en colaboración con actores de la industria.3 La implementación del nuevo etiquetado está programada para finales de 2020. Sin embargo, la nueva regulación enfrenta una oposición feroz por parte de las compañías transnacionales de alimentos y bebidas (CTAB), incluidas Nestlé, Kellogg, Grupo Bimbo, Coca-Cola y PepsiCo a través de sus asociaciones comerciales, la *National Manufacturers Association*, la *American Bakers Association*, la Confederación de Cámaras Industriales de los Estados Unidos Mexicanos (Concamin) y El Consejo Mexicano de la Industria de Productos de Consumo, A.C. (ConMéxico).4-6 México, como el líder regional, podría dar un impulso a favor de replicar el EA en América Latina. Pero el destino del EFNA mexicano y el de la región actualmente radica en la respuesta de este gobierno a tres amenazas de las CTAB que citan tratados internacionales. Nosotros sostenemos que las amenazas de acciones legales que la industria plantea, no deberían impedir que México u otros países implementen políticas basadas en evidencia que persigan objetivos legítimos de salud pública.

La industria amenaza con demandar por violaciones al comercio en la Organización Mundial del Comercio

El Acuerdo de Obstáculos Técnicos al Comercio (OTC) de la Organización Mundial del Comercio (OMC) tiene como objetivo prevenir que las regulaciones, los procedimientos de certificación, las pruebas y las normas (por ejemplo, restricciones de mercadotecnia) planteen obstáculos innecesarios al comercio internacional.7 Las CTAB a través de sus asociaciones comerciales alegan que los cambios al EFNA en México crearán obstáculos innecesarios al comercio y violará el Artículo 2.2 y el Artículo 2.44,5 (Ver tabla).

Esto representa una táctica simple de las CTAB para retrasar la regulación, ya que estas preocupaciones sobre el etiquetado frontal de alimentos se han planteado previamente en el Comité OTC de la OMC para los casos de Chile, Perú, Indonesia (2013), Ecuador (2014) y Uruguay (2019).8 Mientras que el Acuerdo OTC advierte contra cualquier regulación que restringa innecesariamente el comercio, reconoce que cada Miembro de la OMC tiene el derecho básico de proteger la salud humana.7 Los gobiernos han procedido con sus políticas de EFNA y los han justificado ante el Comité OTC alegando que empoderan a los consumidores, aumentan su conocimiento, mejoran la decisión de compra por alimentos más saludables y, así, contribuyen a reducir el riesgo de enfermedades no transmisibles.8

Los CTAB también han argumentado que el EFNA de México restringe a las marcas registradas protegidas a través del Acuerdo sobre los Aspectos de los Derechos de Propiedad Intelectual relacionados con el Comercio de la OMC. De acuerdo con el EFNA, se estipula como requisito que las etiquetas eliminen elementos persuasivos como los personajes de dibujos animados en los empaques de alimentos. Chile procedió con estas medidas en su legislación de etiquetado frontal después de tener éxito utilizando los argumentos aquí mencionados.13 Existen precedentes judiciales que respaldan argumentos similares en relación a los empaques de tabaco: la ley de marcas protege al propietario de la infracción (otros usan su marca registrada), pero no otorga el derecho

de usar la marca comercial en ningún contexto.14,15

Esfuerzos de la industria para aprovechar las normas del Codex Alimentarius

Codex Alimentarius es un programa internacional de normas alimentarias de la Organización de las Naciones Unidas para la Alimentación y la Agricultura (FAO) y la Organización Mundial de la Salud (OMS) que también proporciona directrices internacionales para el etiquetado de alimentos y puede consultarse en foros comerciales. Las normas del Codex son ampliamente aceptadas y a menudo se convierten en normas nacionales 'de facto', pero no deberían ser una barrera para los países que proponen implementar políticas de EFNA más estrictas que las normas del Codex.16

Las CTAB argumentan que la ley del etiquetado frontal de alimentos de México es inconsistente con las normas internacionales (incluido el Codex),4,5 que están reconocidas en el Artículo 2.4 y el Artículo 2.9 del Acuerdo OTC de la OMC (Si una medida no se conforma con las normas internacionales, o no existe una norma relevante, los miembros notificarán a otros miembros, proporcionarán información y darán tiempo para comentarios).7 Aquí, en esencia, las CTAB argumentan que el Codex es el único estándar internacional que habla sobre esta asunto y establece un límite internacional vinculante sobre el rigor de las leyes ya implementadas de EFNA por estados nacionales. Sin embargo, el Codex no provee orientación sobre los detalles de las políticas nacionales.8 De hecho, establece normas voluntarias mínimas sobre las medidas nacionales que garantizan la inocuidad de los alimentos.17 Chile, Perú, Ecuador y Uruguay han avanzado con sus leyes del etiquetado frontal de los alimentos utilizando estos argumentos. Uruguay y México han empleado el Modelo de Perfil de Nutrientes de la Organización Panamericana de la Salud publicado en 2016, así como la evidencia nacional e internacional disponible, para elaborar sus políticas de etiquetado frontal de alimentos.

Esfuerzos para manipular acuerdos comerciales regionales

El capítulo 11 del Tratado de Libre Comercio de América del Norte (TLCAN), un acuerdo comercial regional entre los Estados Unidos, México y Canadá, permitió que inversores extranjeros (por ejemplo, las CTAB) amenazan directamente las regulaciones nacionales, incluyendo las políticas de salud pública que impactan sus inversiones a través de la Solución de Controversias Inversor-Estado en el Centro Internacional de Arreglo de Diferencias.9 Apoyadas en el TLCAN y en otros tratados de inversión extranjera, las CTAB podrían tratar de desafiar la ley del etiquetado frontal de alimentos de México en los tribunales internacionales, aunque es poco probable que estas amenazas tengan éxito basado en los intentos fallidos en el contexto del control del tabaco. En 2010, Philip Morris International demandó a Uruguay por su política que regula las etiquetas de advertencia del tabaco con el argumento de que dicha política resistió un tratado bilateral de inversión. La compañía perdió y terminó pagando $8 millones de dólares por los costos legales de Uruguay.10 De manera similar, Philip Morris Asia demandó al gobierno de Australia por el empaquetado genérico del tabaco argumentando las violaciones al un tratado de inversión bilateral y también perdió, pagando millones en costos.11

En noviembre de 2018, las tres partes del TLCAN renegociaron el tratado, creando El Tratado entre México, Estados Unidos y Canadá (TMEC) que entró en vigor el 1 de julio de 2020, reemplazando el TLCAN. En 2018, los borradores filtrados de un anexo propuesto a la TMEC revelaron una

disposición introducida por los Estados Unidos que habría evitado cualquier símbolo, forma o color de advertencia que 'denote inapropiadamente que existe un peligro al consumir alimentos o bebidas no alcohólicas'.18 Este lenguaje no prosiguió después de que los medios informaran sobre la filtración, aunque dicho intento de bloquear el etiquetado de alimentos subraya cuán agresivamente las CTAB buscan enfriar la propagación de esta política.18 Sin embargo, el lenguaje final del TMEC (Artículo 11.4) permite un lenguaje más amplio que exige reconocer los estándares internacionales relevantes (es decir, directrices del Codex). Esto podría implicar que los Estados Unidos presionen a México en adoptar sus débiles estándares de etiquetado, o los estándares, potencialmente débiles, que establezca en un futuro el Codex.12 No obstante, el nuevo trabajo del Codex se encuentra en las primeras etapas y no necesariamente cada trabajo del Codex termina con la adopción de una norma o directriz; también el trabajo podría suspenderse.

El TMEC parece eliminar la capacidad de las CTAB para desafiar directamente las medidas nacionales en los tribunales internacionales, particularmente aquellas que protegen la salud pública, incluidas las políticas alimentarias, aunque se mantenga el derecho de las corporaciones a desafiar las políticas en otros cinco sectores (petróleo y gas natural, generación de energía, telecomunicaciones, servicios de transporte, y algo de infraestructura).12 Esto reduce aún más la posibilidad de que una amenaza de acción legal en contra del EFNA sea exitosa en México.

Conclusión

Las CTAB están tratando de bloquear el Plan de Acción Mundial para la Prevención y el Control de Enfermedades No Transmisibles 2013-2020 de la OMS, que incluye la implementación de políticas de etiquetado frontal de alimentos interpretativos y obligatorios,19 conteniendo así la réplica de esta innovación de la política de salud pública en México y América Latina. Sin embargo, las acciones legales internacionales resultan ser mucho más fáciles y baratos como herramienta discursiva para amenazar la regulación, que si realmente se llevaran a cabo o si fueran exitosos. Las CTAB prefieren evitar cualquier batalla legal costosa que puedan perder. En el mejor de los casos, las CTAB probablemente retrasarán, pero no bloquearán la implementación del nuevo etiquetado frontal de alimentos mexicano. El poder ejecutivo mexicano ha acelerado la implementación al establecer plazos claros para las pautas de la regulación del EFA, con el apoyo de expertos nacionales e internacionales. México debería continuar con este esfuerzo a pesar de las amenazas vacías de la industria, así como otros países de la región deberán hacer esfuerzos similares.

Este artículo fue traducido por Eric Crosbie y Angela Carriedo.

Tabla. Amenazas de compañías transnacionales de alimentos y bebidas y asociaciones comerciales de acciones legales en contra del EFNA en México: posibles respuestas y evidencia de apoyo

Tratado internacional	Amenazas de acciones legales por las CTAB y asociaciones comerciales	Análisis y respuestas potenciales	Evidencia de apoyo

OTC (OMC)	El EFNA restringe innecesariamente el comercio<br>- Artículo 2.2. (las regulaciones técnicas no deben crear obstáculos innecesarios al comercio).<br>- Artículo 2.4 (cuando existan estándares internacionales aplicables, los Miembros deben usarlos como base para sus regulaciones técnicas, excepto cuando las normas internacionales no alcanzaran de manera efectiva el objetivo legítimo que se haya establecido.)4,5	Mientras que el Acuerdo de Obstáculos Técnicos al Comercio advierte contra cualquier regulación que restringe innecesariamente el comercio, reconoce que cada Miembro de la OMC tiene el derecho básico de implementar medidas para lograr objetivos de políticas legítimos, como la protección de la salud y la seguridad humana.7	- Se plantearon preocupaciones comerciales similares en Chile, Perú e Indonesia en 2013, Ecuador en 2014 y Uruguay en 2019,8 pero estos países avanzaron con su EFNA.<br>- Los estados miembros de la OMC han argumentado a favor del EFNA en discusiones del Comité del OTC de la OMC por 'brindar a los consumidores información suficiente sobre los alimentos que consumen y reducir enfermedades no transmisibles'; 'brindar información a los consumidores para elecciones apropiadas para su dieta y reducir el riesgo de ENT relacionadas a la dieta'; y 'brindar poder a los consumidores a hacer elecciones informadas para fomentar competencia efectiva y el bienestar de los consumidores'.8
ADPIC (OMC)	EFNA restringiría las marcas registradas bajo las ADPIC de la OMC cuando la regulación de etiquetado restringe el uso de elementos persuasivos, como son personajes de dibujos animados, en empaques de alimentos obligados a llevar un etiquetado de advertencia		Se han planteado preocupaciones similares sobre el derecho comercial en Chile, pero sin embargo el país pudo avanzar con el EFNA.8
Codex	El EFNA es inconsistente con los estándares internacionales de la Comisión del Codex Alimentarius (Normas Codex) y los países deben de esperar hasta que el Codex desarrolle las directrices para EFNA.	- No existen directrices del Codex específicas para la disposición de EFNA -dado que el trabajo sobre directrices de EFNA surgió de las provisiones de Codex en torno a información complementaria sobre nutrición- por lo que no es posible ser inconsistente con dichas directrices.	Se hicieron amenazas similares en referencia al Codex anteriormente en Chile y Uruguay, y ambos países siguieron adelante con su etiquetado.

		El Codex establece “normas mínimas” un base y los países pueden optar por exceder estas normas para proteger la salud de sus poblaciones de riesgos a la salud.<br>Si se desarrollan directrices de Codex en el futuro, no es obligatorio cumplir con ellos bajo el derecho comercial.	
TLCAN	El capítulo 11 permite a que inversores extranjeros (por ejemplo, corporaciones) desafiar directamente al EFNA mediante la Solución de Controversias Inversor - Estado a través del Centro Internacional de Arreglo de Diferencias.9<br>- EFNA es una violación de marca debido a la eliminación de gráficas o logos del empaque.	Se presentaron argumentos análogos bajo disposiciones similares en otros acuerdos de inversión en torno al etiquetado de tabaco y tribunales tanto nacionales como legales han fallado contra este argumento ya que la ley de marcas protege al propietario de la infracción (otros que usan su marca registrada), pero no les da derecho a usar la marca registrada en cualquier contexto.<br>- El EFNA también puede justificarse por razones de salud, que puede ser apropiado para limitar el uso de marcas registradas.	En 2010 y 2011 Philip Morris International demandó a los gobiernos de Uruguay y Australia, respectivamente, al impugnar las leyes de empaquetado y etiquetado de tabaco, pero perdió tanto a nivel nacional como internacional y tuvo que pagar millones de dólares en honorarios legales.10,11
TMEC	El artículo 11.4 permite un lenguaje más amplio y coercitivo en el reconocimiento de grupos de estandarización público o privados como estándares internacionales relevantes. Esto puede extenderse a aceptar estándares voluntarios (por ejemplo, estándares corporativos como los de EUA) como equivalentes a los del Codex para el propósito de formular regulaciones nacionales.12	El trabajo actual de Codex sobre EFNA propone orientación general en lugar de un tipo específico de etiquetado. Los procesos del Codex son lentos y el resultado de este trabajo y su eventual estatus legal es todavía incierto.<br><br>Artículo 9.4: Las medidas sanitarias o fitosanitarias que estén en conformidad con normas, directrices y recomendaciones internacionales relevantes se consideran necesarias para proteger la vida y la salud de las personas y de los animales o para preservar los vegetales, y	El TMEC entró en vigor en julio de 2020 pero las guías de EFNA del Codex aún siguen pendientes.

		se presumen compatibles con las disposiciones pertinentes de este Capítulo, del Capítulo 2 (Trato Nacional y Acceso de Mercancías al Mercado), relativas al uso de medidas sanitarias o fitosanitarias, y del Artículo XX (b) del GATT de 1994 según se incorpora en el Artículo 32.1 (Excepciones Generales).<br><br>Artículo 9.6: Cada Parte tiene el derecho a adoptar o mantener las medidas sanitarias y fitosanitarias necesarias para la protección de la vida y la salud de las personas y de los animales	

Siglas: ADPIC, Acuerdo sobre los Aspectos de los Derechos de Propiedad Intelectual relacionados con el Comercio; OTC, Obstáculos Técnicos al Comercio; OMC, Organización Mundial del Comercio; TLCAN, Tratado de Libre Comercio de América del Norte; y TMEC, El Tratado entre México, Estados Unidos y Canadá.

Referencias

1. Taillie LS, Reyes M, Colchero MA, et al. An Evaluation of Chile's Law of Food Labeling and Advertising on Sugar-Sweetened Beverage Purchases from 2015 to 2017: A before-and-after Study. PLoS Med 2020;17:e1003015.
2. Universidad de Chile. Ley De Etiquetado: Cambios en composición de alimentos y de conductas tras su implementación. 21 de noviembre 2018. Disponible en: https://inta.cl/evaluacion-de-panel-de-expertos-nacional-e-internacional-revela-cambios-en-composicion-de-alimentos-y-conductas-de-las-personas-tras-implementacion-de-la-ley-de-etiquetado/. Consultado: 28 de noviembre 2018.
3. De la Cruz-Gongora V, Torres P, Contreras-Manzano A, et al. Understanding and Acceptability by Hispanic Consumers of Four Front-of-Pack Food Labels. Int J Behav Nutr Phys Act 2017;14:28.
4. National Manufacturers Association. Public Submission to Consultation on Mexico Front-of-Pack Nutrition Labeling 10 de diciembre 2019. Disponible en: http://187.191.71.192/respuesta_texto_encuestas/5120. Consultado: 12 de enero 2020.
5. American Bakers Association. Public Submission to Consultation on Mexico Front-of-Pack Nutrition Labeling, 11 de diciembre 2019. Disponible en: http://187.191.71.192/expediente/23561/recibido/63252/B000195554. Consultado: 12 de enero 2020.

6. Krizanovic P. Mexico Food Industry Mulls Legal Fight over New Nutrition Labels. Just Food. 10 February 2020: Disponible en: https://www.just-food.com/news/mexico-food-industry-mulls-legal-fight-over-new-nutrition-labels_id143074.aspx. Consultado: 10 de marzo 2020.
7. World Trade Organization. Technical Barriers to Trade. Marrakech, Marruecos, 15 de abril 1994.
8. Thow AM, Jones A, Hawkes C, et al. Nutrition Labelling Is a Trade Policy Issue: Lessons from an Analysis of Specific Trade Concerns at the World Trade Organization. Health Promot Int 2018;33:561-571.
9. North American Free Trade Agreement. Chapter Eleven: Investment. Nueva York, EE.UU., 1 de enero 1994.
10. Crosbie E, Sosa P, Glantz SA. Defending Strong Tobacco Packaging and Labelling Regulations in Uruguay: Transnational Tobacco Control Network Versus Philip Morris International. Tob Control 2018;27:185-194.
11. Crosbie E, Thomson G, Freeman B, et al. Advancing Progressive Health Policy to Reduce Ncds Amidst International Commercial Opposition: Tobacco Standardised Packaging in Australia. Glob Public Health 2018;13:1753-1766.
12. Labonte R, Crosbie E, Gleeson D, et al. Usmca (Nafta 2.0): Tightening the Constraints on the Right to Regulate for Public Health. Global Health 2019;15:35.
13. Carreño I, Dolle T. The Relationship between Public Health and Ip Rights: Chile Prosecutes Kellogg's, Nestlé and Masterfoods for Using Cartoons Aimed at Attracting Children. Eur J Risk Regul 2017;8:170-177.
14. Crosbie E, Glantz SA. Tobacco Industry Argues Domestic Trademark Laws and International Treaties Preclude Cigarette Health Warning Labels, Despite Consistent Legal Advice That the Argument Is Invalid. Tob Control 2014;23:e7.
15. Crosbie E, Eckford R, Bialous S. Containing Diffusion: The Tobacco Industry's Multipronged Trade Strategy to Block Tobacco Standardised Packaging. Tob Control 2019;28:195-205.
16. Thow AM, Jones A, Huckel Schneider C, et al. Increasing the Public Health Voice in Global Decision-Making on Nutrition Labelling. Global Health 2020;16:3.
17. Cosbey A. A Forced Evolution? The Codex Alimentarius Commission, Scientific Uncertainty and the Precautionary Principle. Winnipeg, Canadá, enero 2000. Disponible en: https://www.iisd.org/sites/default/files/publications/forced_evolution_codex.pdf. Consultado: 1 de diciembre 2019.
18. Ahmed A, Richtel M. In Nafta Talks, U.S. Tries to Limit Junk Food Warning Labels. New York Times. Nueva York, EE.UU. 20 de marzo 2018: Disponible en: https://www.nytimes.com/2018/03/20/world/americas/nafta-food-labels-obesity.html
19. World Health Organization. Follow-up to the Political Declaration of the High-Level Meeting of the General Assembly on the Prevention and Control of Non-Communicable Diseases. Sixty-Sixth World Health Assembly Wha66.10 Agenda Item 13.1, 13.2, 27 de mayo 2013. Annex: Global Action Plan for the Prevention and Control of Noncommunicable Diseases 2013–2020. Ginebra, Suiza, 2013.
